# Risky HIV sexual behaviour and depression among University of Nairobi students

**DOI:** 10.1186/s12991-015-0054-2

**Published:** 2015-04-11

**Authors:** Caleb J Othieno, Roselyne Okoth, Karl Peltzer, Supa Pengpid, Lucas O Malla

**Affiliations:** Department of Psychiatry, University of Nairobi, P.O. Box 19676, 00202 Nairobi, Kenya; ASEAN Institute for Health Development, Mahidol University, Nakhon Pathom, Thailand; Human Sciences Research Council, Pretoria, South Africa; University of Limpopo, Turfloop Campus, Polokwane, South Africa; Kenya Medical Research Institute, Wellcome Trust, Nairobi, Kenya

**Keywords:** Students, Depression, Risky sexual behaviour, HIV

## Abstract

**Background:**

Prevalence rates of human immunodeficiency virus (HIV) infection among the youth are disproportionately high compared to that of other age groups in Kenya. Poor mental health has been linked to risky HIV behaviour, yet few local studies have explored these aspects. This study sought to determine associations between HIV risky sexual behaviour and depression among undergraduate students at the University of Nairobi.

**Method:**

A random sample of 923 (525 males and 365 females) undergraduate students was interviewed using a questionnaire to record sociodemographic variables and risky sexual behaviour including having multiple sexual partners, inconsistent condom use and engaging in sex after drinking. Depressive symptoms were measured using the Centre for Epidemiological Studies Short Depression Scale (CES-D 10).

**Results:**

The students’ mean age was 23 years (s.d.4.0). Overall, 41.33% of the students scored above the cut-off point of 10 on the CES-D 10 scale, with 35.71% having moderate symptoms and 5.62% having severe depressive symptoms. The percentage of those who had ever been diagnosed with sexually transmitted infections (STIs) was 9.71% (males 8.65%; females 11.01%); and for HIV 3.04% (males 2.02%; females 4.05%). Nearly 30% reported having had multiple partners in the previous 12 months, 27.4% of the students did not use condoms with sexual partners and 21% had engaged in sex after drinking within the previous 3 months. In multivariable-bivariate logistic regression, being older, having depressive symptoms, alcohol use/binge drinking, tobacco use, sex after drinking, previous diagnosis of STI, physical abuse, sexual coercion and history of sexual abuse as a child were significantly associated with having multiple partners. Further, younger age, being female, tobacco use and previous diagnosis of STI were significantly associated with inconsistent condom use.

**Conclusion:**

The prevalence of HIV rate infection is low compared to the national average but risky sexual behaviour is common among the students and is positively linked to depressive symptoms among other factors. Programmes aimed at HIV prevention should be integrated with mental health interventions.

## Background

Despite reductions in human immunodeficiency virus (HIV) inflection prevalence rates and increased coverage of those who need ARV drugs in sub-Saharan Africa, the numbers of those infected are still quite high in Kenya and recent studies show a need to reduce the prevalence rate further particularly among the youth [1]. Overall, the national HIV prevalence rate is dropping but wide regional variations continue to be seen. For example, Oluoch and his co-workers [2] reported a national average of 5.6% ranging from 1.2% in North Eastern Province to 16.1% in Nyanza Province. Using data from several sources, recent reports give a national prevalence rate of 6% (5.6% for males and 7.6% for females). The range varied from 0.2% in Wajir in the northern parts to 25.7% in Homa Bay County around Lake Victoria. Although the HIV prevalence among the youth is 2.7% for females and 1.7% for males, the report estimated that young women in the age group 15–24 years account for 21% of all the new HIV infections in Kenya [3].

The high rates of HIV infection in the youth could be linked to risky sexual behaviour since most of the HIV infections are through heterosexual transmission. For example, inconsistent condom use and unprotected sex with multiple partners, both of which are risk factors for HIV, have been reported among university students [4-7]. A study involving 24 countries in sub-Saharan Africa reported that multiple sexual partnerships were more common among urban males and females with higher education [8]. Among university students, the changes in living arrangements and access to information further increase these risks. [5,9]. Since the university population is a vulnerable group with respect to HIV, it is important to determine factors related to the risky behaviours [5,10,11] and to monitor the trends [8].

Depressive symptomatology in youth has been associated with high-risk sexual behaviour such as early sexual debut, higher number of sexual partners and having sex while under influence of alcohol and drugs [12]. Similar findings have been demonstrated in studies done in sub-Saharan Africa where HIV is endemic in several countries. Youths in Uganda who had high scores on depression were more likely to report having high numbers of sexual partners [13]. In South Africa, there is evidence that depression is linked to risky sexual behaviour. Young men and women were more likely to have experienced intimate partner violence and to have engaged in unprotected sex or to report incorrect condom use [14]. The same study also found that depression could be a marker of increased HIV risk [14]. The same trends have been observed among university students in Ivory Coast where poor mental health including alcohol use and partner violence was found to be associated with HIV risk behaviour [15]. Although studies from other parts of the world have linked poor mental health to risky sexual behaviour, relatively few studies have been done in Kenya on adolescents and youth mental health. This study, therefore, aims to provide more data on the links between depression and HIV risky behaviour.

### Objective

The objectives of this study were to describe the HIV risky sexual behaviours in relation to depressive symptoms and other sociodemographic variables among undergraduate students at the University of Nairobi in Kenya.

## Methods

### Study population

We obtained permission from the Kenyatta National Hospital and the University of Nairobi Ethics and Research Committee. University of Nairobi is the largest of the seven public universities in Kenya. It has six colleges; College of Architecture and Engineering (Main Campus), College of Humanities and Social Sciences (Main Campus), College of Health Sciences (Kenyatta National Hospital), College of Education and External Studies (Kikuyu Campus and Kenya Science Campus), College of Agriculture and Veterinary Sciences (Upper Kabete Campus) and the College of Biological and Physical Sciences (Chiromo Campus). The research targeted the University of Nairobi students. The total student population in the University of Nairobi is 36,991 (22,734 males and 14,257 females) [16].

### Sampling design

From the total registered students in the University of Nairobi, each of the six colleges participated in the study in order to achieve representativeness and to increase statistical power. Proportional stratified sampling was used to ensure that the colleges forming different student subpopulations were represented in the sample in same proportions as the population. The sampling process proceeded in two steps. In the first stage, the number of participants to be obtained from each college was determined using calculations based on probability proportional to size. Next, a sampling frame containing a list of all students was compiled from each college. In the second step, a simple random sample was selected within each college using computer-generated random numbers and the available sampling frame.

### Measurement of study variables

We administered a purpose-designed questionnaire to record sociodemographic data including age, sex year of study, socioeconomic status and performance in studies.

Questions on risky sexual behaviour enquired on having multiple sexual partners, inconsistent condom in past 3 months, sex after drinking alcohol, age at first pregnancy/made someone pregnant and past history of sexually transmitted infection.

#### Depression

The Centre for Epidemiological Studies Short Depression Scale (CES-D 10) consists of ten questions. It has been used in other parts of sub-Saharan Africa. We used a cut-off point of ≥10. Those who had a score of 0–9 were classified as having a mild level of depressive symptoms, 10–14 as moderate depressive symptoms and ≥15 representing severe depressive symptoms. [17-19]. Binge drinking was defined as having more than four or five drinks at a sitting.

#### HIV risk behaviour

In terms of HIV risk behaviour, university students were asked, “During the past 12 months how many sexual partners did you have?” “During the past 3 months did you use a condom with your primary partner?” (Response options ranged from 1 = never to 5 = every time; inconsistent condom use was defined as not having used a condom every time in the past 3 months). Further, students were asked if they had had sex after drinking alcohol in the past 3 months, with the response option, “yes” or “no”.

*STIs and HIV status*: STIs status was assessed with the question, “Have you ever been diagnosed with a sexually transmitted infection?” and HIV status, “Have you ever been diagnosed HIV positive?

### Handling of survey non response and missing data

The response rate was about 70%. To achieve this, extensive awareness of the research was done and this included visits of lecture halls, by research assistants, to educate students on the benefits of the study and also the use of study introductory letter on every questionnaire. Research assistants traced the selected students through the chairmen of their departments where they obtained their contacts. Thereafter, they rung the students to introduce the study and arrange a meeting. Those who were unreachable on phone were traced through their colleagues and their lecture halls. All the respondents filled in a pencil-paper-based questionnaire. All missing data were assumed to be at random.

### Statistical analysis

Results of sociodemographic factors were presented using frequencies and proportions, with their distributions examined separately for males and females. Also, the prevalence of depressive symptoms was presented using proportions, and association between depressive symptoms and potential predictors was investigated using chi-square test. On the other hand, a bivariate logistic regression was fitted to determine the associations between risky sexual behaviour, defined by inconsistent condom use and multiple sexual partners, and potential mental health covariates. Variable selection proceeded as follows; first, unadjusted bivariate model was fitted for each covariate. All covariates that were statistically significant (*P* value <0.05) were then included in a multivariable-bivariate model. In the study design, sampling was clustered within colleges and college membership included as a fixed effect in the final bivariate model. Twelve respondents, who were married and did not use condoms with partners, were excluded in bivariate analyses.

#### Software

Exploratory analyses, calculation of frequencies and proportions were done using IBM SPSS (version 22.0) and bivariate logistic regression fitted using the Zelig package in R version 3.0.2.

## Results

The sociodemographic characteristics of the study sample are shown in Table 1. We obtained data from 923 students (525 males and 365 females). The mean age was 23 years (s.d.4.0). Two-thirds resided within the campus. Ninety percent were single. More females (15%) were married compared to the males (approximately 7%). Nearly half (48%) of the students rated themselves as coming from families that were either not well off or were poor. Less than 1% of the students recorded their academic performance as not satisfactory. The percentage of those who had ever been diagnosed with sexually transmitted disease was 9.71% (males 8.65%; females 11.01%); and for HIV 3.04% (males 2.02%; females 4.05%).

**Table 1:** **Sociodemographic characteristics**

	**Overall**		**Male**		**Female**	
	***N***	**%**	***N***	**%**	***N***	**%**
All	923		525		365	
Age						
<20	200	23.39	122	24.11	78	22.35
20–24	515	60.23	311	61.46	204	58.45
25–29	84	9.82	49	9.68	35	10.03
30 and above	56	6.54	24	4.74	32	9.17
Year of study						
*First*	185	21.17	128	25.40	56	15.60
*Second*	256	29.29	143	28.37	111	30.92
*Third*	195	22.31	107	21.23	85	23.68
*Fourth, fifth and sixth*	238	27.22	126	25.00	107	29.81
College						
CAVS	66	7.17	41	7.69	25	6.70
CAE	149	16.18	101	18.95	45	12.06
CBPS	150	16.28	93	17.45	57	15.28
CEES	193	20.95	109	20.45	81	21.72
CHS	30	3.25	12	2.25	18	4.83
CHSS	333	36.16	177	33.21	147	39.41
Marital status						
Married	90	9.96	35	6.67	54	14.79
Single	813	90.03	490	93.33	311	85.21
Religion						
Christian	752	82.28	439	82.67	303	82.34
Muslim	61	6.67	34	6.40	24	6.52
Other	101	11.05	58	10.92	41	11.14
Residence						
On campus	616	67.99	387	73.71	219	60
Off campus (on your own or with parents/guardians)	290	32.01	138	26.28	146	40.11
Family background						
Wealthy/Quite well off	469	52.00	251	47.68	207	56.87
Not very well off/Quite poor	433	48.0	271	51.91	157	43.13
Academic performance						
Excellent/Very good	449	57.93	260	57.14	182	58.30
Good/Satisfactory	320	41.29	191	41.97	125	40.46
Not satisfactory	6	0.77	4	0.88	2	0.65

### Depression

The prevalence of depression among the students is shown in Table 2. Overall, 41.33% of the students scored above the cut-off point of 10 on the CES-D 10 scale, with 35.71 having moderate symptoms and 5.62% having severe depressive symptoms. Proportionately, more females had depressive symptoms compared to males but the difference was not statistically significant. Similarly, no statistically significant difference was noted among the different age groups although approximately half of those in the age group of 30 years and above were reported to have mild–moderate symptoms of depression. The rates of depressive symptoms among those who reported binge drinking were high (though not statistically significant) compared to that of those who did not report binge drinking. However, tobacco use was significantly associated with depressive symptoms.

**Table 2:** **Prevalence of depressive symptoms**

	**Moderate (%)**	**Severe (%)**	**Chi-square (** ***P*** **value)**
All	35.71 (30.03–36.20)	5.62 (3.90–6.89)	
Social demographic			
Age			
<20	30.65 (24.22–37.89)	6.45 (3.53–11.26)	0.0933
20–24	37.01 (32.71–41.51)	5.82 (3.97–8.40)	
25–29	27.50 (18.39–38.80)	6.25 (2.32–14.61)	
30 and above	50.94 (37.00–64.75)	1.89 (0.00–11.38)	
Gender			
Male	33.54 (29.39–37.96)	5.35 (3.59–7.84)	0.2598
Female	39.03 (33.94–44.37)	5.13 (3.16–8.13)	
Year of study			
*First*	36.26 (29.15–43.98)	7.60 (4.28–12.92)	0.2598
*Second*	35.74 (29.69–42.27)	4.26 (2.18–7.92)	
*Third*	38.33 (31.28–45.89)	5.56 (2.85–10.26)	
*Fourth, fifth and sixth*	33.63 (27.54–40.29)	4.48 (2.29–8.33)	
College			
CAVS	35.00 (23.44–48.48)	6.67 (2.16–17.00)	0.0745
CAE	38.24 (30.15–46.99)	6.62 (3.26–12.55)	
CBPS	34.33 (26.48–43.09)	5.22 (2.31–10.87)	
CEES	42.62 (35.42–50.14)	2.73 (1.01–6.60)	
CHS	14.81 (4.86–34.61)	0.00	
CHSS	33.33 (28.18–38.90)	7.37 (4.83–11.00)	
Marital status			
Married	37.21 (27.22–48.35)	4.65 (1.50–12.13)	0.9073
Single	35.56 (32.14–39.13)	5.61 (4.12–7.58)	
Religion			
Christian	36.55 (32.98–40.26)	5.04 (3.58–7.00)	0.3581
Muslim	29.09 (18.02–43.09)	7.27 (2.36–18.43)	
Other	33.68 (24.51–44.20)	9.47 (4.69–17.67)	
Residence			
On campus	35.27 (31.37–39.38)	6.70 (2.36–18.43)	0.0833
Off campus (on your own or with parents and guardians)	35.93 (30.26–42.00)	2.96 (1.38–5.98)	
Family background			
Wealthy/Quite well off	33.72 (29.30–38.43)	2.56 (1.35–4.67)	<0.0001
Not very well off/Quite poor	37.44 (32.75–42.37)	8.87 (6.37–12.17)	
Academic performance			
Excellent/Very good	37.23 (32.62–42.08)	5.97 (3.98–8.79)	0.1754
Good/Satisfactory	30.17 (25.06–35.81)	5.76 (3.50–9.24)	
Not satisfactory	50.0 (18.76–81.23)	16.67 (0.87–63.52)	
Alcohol usage			
Alcohol use	38.9 (33.78–44.28)	5.76 (3.65–8.90)	0.2473
No Alcohol use	33.53 (29.46–37.85)	5.52 (3.78–7.98)	
Binge drinking			
Binge drinking	41.11 (33.92–48.69)	6.11 (3.24–10.95)	0.3160
Non-binge drinking	35.90 (27.39–45.35)	3.42 (1.10–9.04)	
Tobacco usage			
Tobacco use	37.63 (27.97–48.33)	13.98 (7.94–23.08)	0.0004
Non-tobacco use	33.83 (30.08–37.80)	4.50 (3.04–6.56)	
Sexual behaviour			
Number of partners for past 12 months			
None or 1	34.15 (30.29–38.24)	4.40 (2.93–6.52)	0.0195
≥2	38.81 (33.18–44.75)	8.04 (5.28–11.98)	
Consistent use of condom with partner	32.78 (28.37–37.51)	6.84 (4.71–9.79)	0.0967
Non–consistent use	38.60 (34.01–43.41)	4.42 (2.76–6.94)	
Sex after drinking past 3 months	46.75 (39.09–54.55)	5.33 (2.22–9.43)	0.0066
No sex after drinking past 3 months	33.50 (29.76–37.46)	4.73 (3.73–7.52)	
Ever diagnosed with STI	50.65 (39.10–62.13)	6.49 (2.42–15.15)	0.0101
Never diagnosed with STI	34.26 (30.82–37.87)	5.13 (3.69–7.07)	
Ever diagnosed with HIV	57.69 (37.19–76.03)	19.23 (7.31–39.98)	<0.0001
Never diagnosed with HIV	34.58 (31.25–38.05)	4.88 (3.52–6.71)	
Ever been hit by a sexual partner	56.67 (43.30–69.18)	11.67 (5.21–23.18)	<0.0001
Never been hit by sexual partner	34.05 (30.67–37.59)	4.67 (3.34–6.53)	
Ever been forced to have sex	65.12 (54.00–74.87)	9.30 (4.39–18.00)	<0.0001
Never been forced to have sex	32.28 (30.11–37.10)	4.83 (3.43–6.72)	
Physically abused as a child	43.24 (31.94–55.24)	13.51 (7.02–23.91)	0.0007
Never physically abused as a child	34.83 (31.41–38.40)	4.63 (3.27–6.47)	
Sexually abused as a child	53.06 (38.42–67.22)	8.16 (2.65–20.48)	0.0122
Never sexually abused as a child	34.56 (31.20–38.09)	5.15 (3.73–7.01)	

Other factors associated with high levels of depressive symptoms included having a positive history of HIV or STI infection. Those who reported traumatic events such as having been hit by a sexual partner, having been forced to have sex and having been physically abused as a child also reported significantly higher levels of depressive symptoms compared to those who did not report such events.

### Association with risky sexual behaviour

The two factors defining risky sexual behaviour were having more than two sexual partners within the past 12 months and inconsistent condom use. The former was reported by 30% of the students. Inconsistent condom use was reported by 27.48% of the students (males 26.74 and females 28.75). One-fifth of the students reported engaging in sex after drinking. In multivariable-bivariate logistic regression, being older, having depressive symptoms, alcohol use/binge drinking, tobacco use, sex after drinking, previous diagnosis of STI and sexually abused as a child were significantly associated with having multiple partners. Further, younger age, being female, tobacco use and previous diagnosis of STI were significantly associated with inconsistent condom use (Table 3).

**Table 3:** **Associations between risky sexual behaviour (multiple sexual partners, inconsistent condom use) and mental health covariates**

	**Multiple sexual partners**		**Inconsistent condom use**	
	**Unadjusted odds Ratio (95% C.I)**	**Adjusted odds ratio (95% C.I)**	**Unadjusted odds ratio (95% C.I)**	**Adjusted odds ratio (95% C.I)**
Social demographic				
Age	0.95 (0.92–0.99)**	1.12 (1.00–1.28)*	0.85 (0.80–0.89)***	0.90 (0.83–0.97)***
Gender				
Female^a^				
Male	1.74(1.30–2.33)***	4.10 (1.72–10.43)	0.87 (0.66–0.92)*	0.99 (0.67–1.47)***
Year of study				
*First* ^a^				
*Second*	1.36 (0.92–2.03)	3.20 (0.93–11.69)	0.89 (0.61–1.31)	-
*Third*	1.26 (0.82–1.93)	2.18 (0.58–8.56)	0.74 (0.49–1.12)	-
*Fourth, fifth and sixth*	1.76 (1.17–2.67)**	3.05 (0.84–11.61)	0.80 (0.54–1.19)	-
College				
CAVS^a^				
CAE	0.87 (0.43–1.67)	0.29 (0.04–2.06)	0.61 (0.33–1.11)	1.22 (0.52–2.86)
CBPS	0.54 (0.27–1.03)	0.99 (0.12–7.37)	0.40 (0.22–0.73)**	0.55 (0.23–1.31)
CEES	0.36 (0.19–0.67)**	0.52 (0.34–0.62)*	0.24 (0.13–0.43)***	0.46 (0.18–1.13)
CHS	0.76 (0.29–2.05)	0.31 (0.03–2.55)	1.28 (0.51–3.36)	2.71 (0.76–10.46)
CHSS	0.73 (0.39–1.33)	0.67 (0.05–10.70)	0.63 (0.36–1.08)	0.76 (0.34–1.66)
Marital status				
Married^a^				
Single	1.08 (0.66–1.75)	-	1.56 (0.98–1.92)	-
Religion				
Christian^a^				
Muslim	1.53 (0.85–2.90)	-	0.66 (0.38–1.14)	-
Other	1.16 (0.75–1.85)	-	0.60 (0.39–1.12)	-
Residence				
On campus^a^				
Off campus (on your own or with parents and guardians)	1.18 (0.87–1.60)	-	0.47 (0.35–1.42)	-
Family background				
Wealthy/Quite well off^a^				
Not very well off/Quite poor	1.10 (0.83–1.45)	-	1.05 (0.80–1.37)	-
Academic performance				
Excellent/Very good^a^				
Good/Satisfactory	0.94 (0.70–1.28)	-	1.49 (0.07–1.99)	-
Not satisfactory	1.03 (0.20–7.50)	-	0.56 (0.08–2.90)	-
Depression				
No depression^a^				
Moderate	1.78 (1.57–2.05)	2.76 (1.83–3.11)**	1.80 (1.60–2.06)*	0.92 (0.61–1.39)
Severe	1.51 (1.27–1.95)*	2.01 (1.50–3.22)**	1.38 (0.75–2.59)	1.18 (0.47–2.97)
Alcohol use **(**reference = No**)**	2.07 (1.57–2.75)***	3.68 (2.64–4.13)***	1.55 (1.18–2.02)**	1.98 (0.64–2.52)
Binge drinking (reference = No)	1.82 (1.14–2.89)*	2.12 (1.85–2.46)**	1.18 (0.74–1.89)	-
Tobacco use (reference = No)	3.36 (2.18–5.22)***	2.26 (2.02–2.39)***	1.97 (1.27–3.14)**	1.78 (1.44–2.87)**
Sexual behaviour				
Sex after drinking past 3 months (reference = No)	5.10 (3.58–7.33)***	3.94 (1.62–10.17)*	3.81 (2.59–5.72)***	3.77 (0.22–7.04)
Ever diagnosed with STI (reference = No)	3.70 (2.32–5.99)***	6.48 (1.36–20.33)*	3.60 (2.12–6.44)***	2.99 (1.21–4.50)*
Ever diagnosed with HIV (reference = No)	2.25 (0.97–5.25)		2.53 (1.04–7.09)	-
Ever been hit by a sexual partner (reference = No)	2.33 (1.32–3.77)**	2.35 (0.44–3.77)	2.39 (0.33–4.36)	2.13 (0.11–6.23)
Ever been forced to have sex (reference = No)	1.74 (1.11–2.73)*	3.56 (0.92–4.61)	1.68 (1.07–2.69)*	5.29 (0.43–17.50)
Physically abused as a child (reference = No)	1.04 (0.63–1.67)	-	1.01 (0.64–1.61)	-
Sexually abused as a child (reference = No)	1.78 (0.98–3.20)	1.87 (1.52–2.11)**	2.20 (1.19–4.30)*	1.81 (0.92–3.21)

Based on bivariate logistic regression estimates.

*P < .05; **P < .01; ***P < .001.

^a^Reference level.

C.I = confidence level.

## Discussion

The overall prevalence rate of HIV in this sample was 3.04% (males 2.02% and females 4.05%). This is lower than the Kenyan national average for a comparable age group. However, the study shows that a considerable number of students engage in risky sexual behaviour. Approximately 30% reported having had multiple sexual partners, and one-fifth of the students had engaged in sex after drinking. More than one quarter of both males and females reported non-use of condoms with sexual partners, yet 36% of the males and 21% of the females had more than two sex partners in the proceeding 12 months. These findings are comparable to other studies among university students in the region. A local study among Maseno University students in Western Kenya reported that 25.6% of the students engaged in sex while intoxicated with alcohol [20]. Similar findings have been also been reported from Ugandan university students where risky sexual behaviour was linked to drinking and high STI levels [21].

HIV transmission in Kenya is mainly through heterosexual sex, and efforts to decrease the HIV transmission rates in Kenya have concentrated mainly on reproductive health with an emphasis on safe sexual practices such as condom use, voluntary counselling and testing and more recently voluntary medical male circumcision [22]. While these interventions have had some effect and there has been a general decline in HIV prevalence rates among the age group 15–49 from 1990s to 2008, there are concerns that the prevalence rate has since stabilised and that infection rates among the youth account for 21% of all new infections [3]. To sustain the gains made, a rate of zero infections should be the aim. It is therefore essential to explore all the correlates of risky sexual behaviour and formulate appropriate interventions.

Risky sexual behaviour is positively linked to depression as has been shown in studies from other student groups [13,14]. Similarly in this study, having depressive symptoms was associated with having multiple partners. It could be that depressive feelings lead to risky sexual behaviour. Alternatively, risky sexual behaviour, being forced to have sex and being hit by a sexual partner, factors which are high in this sample, are the causes of high depressive symptoms. Sexual coercion was also found to be common among Ugandan university students, having affected nearly one-third of those sampled and was associated with subsequent risky sexual behaviour [23]. Further studies are needed to confirm these links particularly in areas with high HIV prevalence rates.

Given the low number of mental health workers in Kenya, it is possible that many of those with moderate to severe depressive symptoms that would need some intervention may go untreated. It is therefore important that workers in reproductive health or general health facilities be made aware of the links between STI risky HIV behaviour and poor mental health. Students who present to the student health services with STIs, history of alcohol use and physical abuse should be screened for depressive illnesses. Programmes that address issues of HIV, for example awareness campaigns, should also include discussions on identification of depression.

### Limitations

Since this was a cross-sectional study dependent and was dependent upon self report of symptoms, there could have been errors related to inaccurate reporting. The university population sampled in this study also differs from the youth population in education and exposure to different cultures and social pressures. These factors could limit the generalisability of the results. However, this is a national university that admits students from all over Kenya and so the findings may be applicable to students from other developing countries in the region.

## Conclusions

HIV rates are lower in the students’ population compared to the national average, but the prevalence of risky sexual behaviour is high and positively linked to depression. Interventions aimed at HIV prevention should be integrated with mental health assessment and treatment.
